# Impact of Hip Rotation Angle Following Total Hip Arthroplasty with Leg Lengthening

**DOI:** 10.3390/jcm14051564

**Published:** 2025-02-26

**Authors:** Norio Imai, Yuki Hirano, Daisuke Homma, Yuki Komuta, Yoji Horigome, Hiroyuki Kawashima

**Affiliations:** 1 Division of Comprehensive Musculoskeletal Medicine, Niigata University Graduate School of Medical and Dental Sciences, 1-757, Asahimachi-dori, Chuo-ku, Niigata City 951-8510, Japan; 2 Division of Orthopedic Surgery, Department of Regenerative and Transplant Medicine, Niigata University Graduate School of Medical and Dental Sciences, 1-757, Asahimachi-dori, Chuo-ku, Niigata City 951-8510, Japan

**Keywords:** total hip arthroplasty, hip rotation angle, leg lengthening, global femoral offset, femoral version

## Abstract

**Background/Objectives**: Few studies report on hip rotation after total hip arthroplasty (THA); however, details of the factors affecting the hip rotation angle are unknown. We aimed to investigate the factors related to hip rotation after THA. **Methods**: This study included 124 consecutive patients who underwent THA. We retrospectively analyzed the correlation between changes in the rotation angle of the femur relative to the pelvis, global femoral offset, and femoral version and leg lengthening. Moreover, we performed a multivariate regression analysis of these parameters to calculate the efficacy of the change in the rotation angle of the femur relative to the pelvis. **Results**: Leg lengthening and femoral version change were negatively correlated, whereas change in global femoral offset was positively correlated with leg lengthening, with correlation coefficients of 0.376, 0354, and 0.334, respectively. Regarding the multiple regression analysis, only leg lengthening was correlated with the change in rotation angle of the femur relative to the pelvis, with a coefficient of −0.336. **Conclusions**: The change in the rotation angle of the femur relative to the pelvis is only associated with leg lengthening in multivariate analysis. In actual planning, in cases where the hip is internally rotated, it may be better not to excessively increase leg length, decrease anterior stem anteversion, or increase global femoral offset. Thus, physicians should avoid large leg lengthening for patients with highly external rotation in their hip joint as it may lead to increased internal rotation of the hip, consequently resulting in relative malpositioning and subsequent implant impingement and/or dislocation following THA.

## 1. Introduction

The implantation angle is important to prevent dislocation and prolong the durability of total hip arthroplasty (THA) [1,2,3,4,5,6]. Various reports have described the optimal implantation angle [1,3,7]. In recent years, the acetabular component is placed not only in relation to the anatomical reference plane of the pelvis but also in consideration to the anteroposterior tilt of the pelvis in the supine and standing positions [8]. This is because pelvic tilt can affect the functional anteversion and inclination of the acetabular component, thereby increasing the likelihood of edge loading and impingement which can affect the postoperative outcomes of implants [9,10]. In addition, pelvic tilting and the extent of change vary widely among individuals [11,12,13], and considering these factors in the placement of implants is important [11,12,13,14].

However, few studies have reported on hip rotation, particularly radiographic rotation based on CT reconstructions, rather than clinical or functional rotation measured via goniometry or motion analysis after THA [15,16,17]. The specific factors affecting the hip rotation angle are unknown as hip rotation cannot be measured using plain radiographs, but it can be measured with CT or special equipment such as sterEOS [17,18,19,20]. Understanding the characteristics of and factors contributing to changes in hip rotation associated with THA is important because hip rotation also changes the functional anteversion angle of the stem, which can be a factor in implant-to-implant impingement and, ultimately, dislocation or implant fracture.

We aimed to investigate the factors related to hip rotation after THA. Based on previous studies, we hypothesized that hip rotation after THA would be affected more by the extent of leg lengthening than by the anteversion angle of the stem. This is because the artificially replaced hip joint, which does not have any sensory system, is unable to adjust its rotation by itself.

## 2. Materials and Methods

### 2.1. Participants

This retrospective study was conducted using preoperative and postoperative computed tomography (CT) images and medical patient records.

This study included THA performed at our institution between 1 April 2010, and 30 September 2022. Overall, 371 patients underwent THA during the study period. We included the following patients: (1) patients who were involved only on the surgical side with hemilateral hip osteoarthritis (HOA) and (2) patients whose center-edge angle was <25° on the nonsurgical healthy side without any symptoms. However, we excluded the following patients: (1) patients who had undergone any surgery on the THA side, (2) patients with any symptoms at the thoracic and lumbar spine or at their knee or ankle, (3) patients with knee osteoarthritis (OA) at grades 2–4 evaluated in the Kellgren–Lawrence classification, or (4) patients with subluxation in the THA site evaluated as Crowe type 3 or 4 on plain radiography. Finally, 124 consecutive patients (93 females and 31 males) who completed the survey were included in the current study. All THAs in this study were performed by five trained orthopedic surgeons through the anterolateral approach in the supine position [21,22,23]. The acetabular components were aimed at placing the center of the acetabular component close to the original hip center at 40° of inclination and 15° of anteversion, relative to the functional pelvic plane (FPP) [24]. The tilt in the sagittal plane of the anterior pelvic plane included the top of the pubic symphysis and bilateral superior iliac spine in the supine position [25]. All acetabular components were placed with the assistance of a mechanical guide [26] or CT-based navigation [27], and the implantation accuracies were within 2° and 3°, respectively, relative to preoperative planning [26,27]. A high hip center (<10 mm) was accepted. The femoral component was anteverted 10–30° relative to the retrocondylar plane (RCP), which contains the most posterior points of the greater trochanter and bilateral femoral condyles [28], such that the sum of the anteversion of the acetabular and femoral components, combined with anteversion, is approximately 30–40° [1,29]. The leg length difference was planned to be equal on both sides and placed according to the preoperative plan; however, the final neck length was determined according to soft tissue balance during surgery. Intraoperative fluoroscopy was not used to confirm offset and leg length discrepancies during the study period.

The study protocol was approved by the ethics review committee (2024-0252). As this was a cross-sectional retrospective study without intervention, we waived the requirement for written informed consent and used the opt-out method.

### 2.2. Measurement

**Figure 1:**
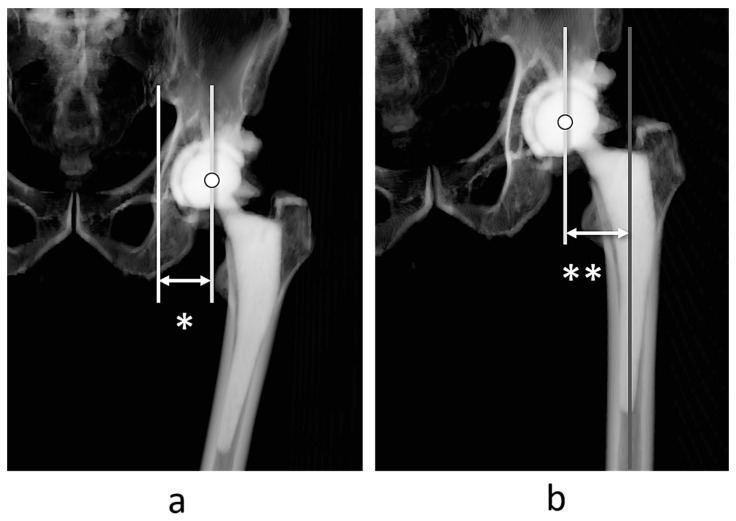
Measurement of acetabular and femoral offset. Acetabular offset is defined as the distance between the femoral head center and teardrop * (**a**). The femoral offset is defined as the distance between the femoral head center and the axis of the proximal femur ** (**b**).

We constructed a three-dimensional (3D) bone model of the pelvis and femur using ZedView^®^ software [30,31,32] version 18.0.0 (Lexi, Tokyo, Japan) using CT images taken for 3D planning before THA and the evaluation of implant positioning 1 week after THA [27,33]. First, after reconstruction of the 3D pelvis model, we aligned it to the anterior pelvic plane and measured the acetabular offset and distance from the teardrop to the center of the femoral head (Figure 1a).

Similarly, the 3D femur model was aligned with the RCP. Subsequently, the femoral offset, the distance between the center of the femoral head to the axis of the proximal femur (Figure 1b), and then the global femoral offset (GFO) were calculated as the sum of the acetabular and femoral offset [33].

Finally, the FPP and RCP were aligned parallelly [34,35]; therefore, the GFO in this study was considered unaffected by the hip position, such as abduction/adduction and internal or external rotation.

**Figure 2:**
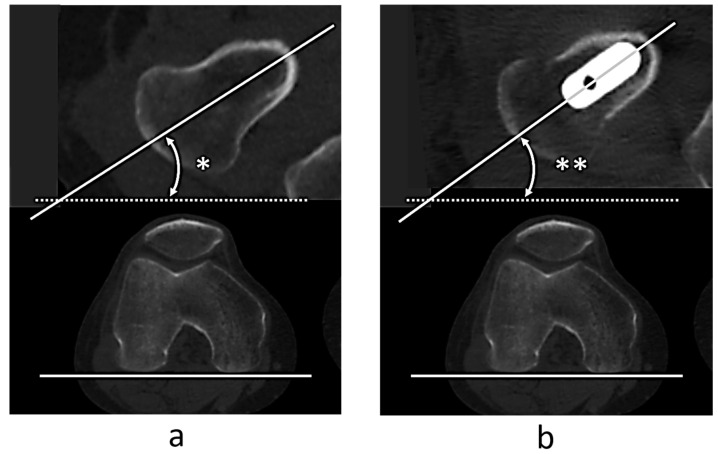
Measurement of femoral version. Femoral neck anteversion * is defined as the angle between the neck axis and the retrocondylar plane (RCP) before total hip arthroplasty (THA) (**a**). (**b**) Stem anteversion ** defined as the angle between the stem axis and RCP after THA.

Regarding the femoral version (FV), we examined the femoral neck anteversion (FNA) using the original femur before surgery and the stem anteversion using the implanted femur after surgery using CT images aligned in the femoral coordinate system. We defined the femoral neck axis as the centerline between the anterior and posterior margins of the femoral neck in the axial plane just below the femoral head, according to Sugano’s method [36]. We defined the angle between the femoral neck axis and the RCP as the FNA (Figure 2a). Similarly, we defined the angle between the stem axis and RCP as the stem anteversion angle and measured it in the same coordinate system as that before surgery (Figure 2b).

ΔFV was defined as the difference between the FNA and stem anteversion.

**Figure 3:**
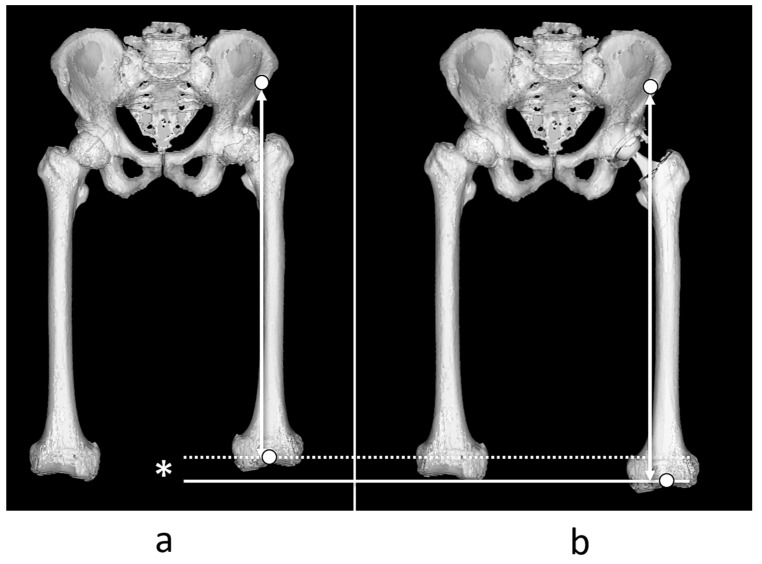
Measurement of leg lengthening. Leg lengthening was defined as the discrepancy between the distance from the anterior superior iliac spine and the knee center point before total hip arthroplasty (THA) (**a**) and after THA (**b**) *.

Leg lengthening was defined as the difference in distance between the anterior superior iliac spine of the surgical site and the most distal point of the intercondylar fossa of the femur in the femoral coordinate system before and after THA (Figure 3).

**Figure 4:**
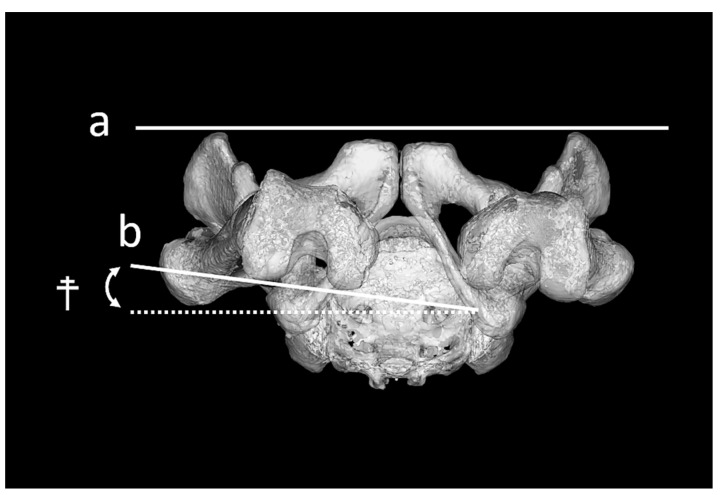
Measurement of the angle of the functional pelvic plane-retrocondylar plane (FPP-RCP). The angle the FPP-RCP ^☨^ was defined as the angle between the functional pelvic plane (a) and retrocondylar plane of the femur (b).

The hip rotation angle was evaluated by internal and external rotations of the femur relative to the pelvis, based on previous studies [15,16]. RCP relative to FPP (FPP-RCP) was defined as the angles between RCP projected onto the transverse plane of the FPP (Figure 4). FPP-RCP was measured as the angle of hip rotation before and after the surgery.

In this study, external rotation of the RCP relative to the FPP was described as positive and internal rotation as negative.

The differences between pre- and postsurgical GFO, FV, and FPP-RCP are expressed as ΔGFO, ΔFV, and ΔFPP-RCP, respectively.

Length measurements, such as GFO and leg lengthening, were corrected per 100 cm in height for ease of calculation in actual planning as a body size correction. The angle of the acetabular component was measured relative to FPP.

All participants were examined using the modified Harris hip score (mHHS) by a skilled orthopedic surgeon within 2 months before and 1 year after THA.

### 2.3. Statistical Analyses

SPSS software version 28 (SPSS Inc., Chicago, IL, USA) was used for the statistical analyses. First, linear regression was analyzed between the operative side GFO, ΔFV, leg lengthening, and ΔFPP-RCP, according to previous reports that described values of <0.2 as no correlation; 0.2 to <0.4 as a weak correlation; 0.4 to <0.7 as a moderate correlation; and ≥0.7 as a strong correlation [37,38,39]. Moreover, we also performed multivariate regression analysis among these parameters to calculate the effectiveness to ΔFPP-RCP. The independent factor was ΔFPP-RCP, whereas the dependent factors were age, sex, body mass index, ΔGFO, ΔFV, and leg lengthening. We also included preoperative FPP-RCP because that could be affected by ΔFPP-RCP. For correlation, we performed a post hoc analysis for statistical power (type II [β] error), with 0.5 as an effect size (d) and 0.05 as type I (β) error. Intra-observer reliability was assessed by one observer measuring all parameters in all patients at least twice within an interval of >4 weeks. Inter-observer reliability was also examined by two observers using all parameters in all patients by the intraclass correlation coefficient (ICC). *p* < 0.05 was considered statistically significant.

## 3. Results

### 3.1. Demographic Data

The participants comprised 93 females and 31 males, aged 57.6 years on average (Table 1).

The body mass index was 24.4 kg/m^2^ on average (Table 1). More than 80% of the participants underwent THA via secondary HOA due to developmental dysplasia of the hip (Table 1). The acetabular cup was placed 40.6 ± 4.7° at inclination and 17.1 ± 6.3° at anteversion. The mHHS significantly improved from 48.8 to 90.2.

### 3.2. Measurements and Statistical Analyses

The GFO was −25 mm shortened, although the leg was 10 mm lengthened (Table 2).

**Figure 5:**
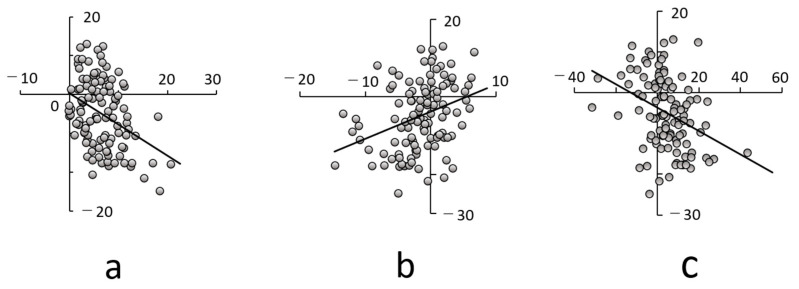
Correlation between Δ functional pelvic plane (FPP), retrocondylar plane (RCP) leg lengthening, Δ global femoral offset (GFO), and Δ femoral version (FV). There was a negative correlation between ΔFPP-RCP and leg lengthening (**a**) and ΔFV (**c**), and a positive correlation was observed between ΔFPP-RCP and ΔGFO (**b**).

The FV was 3.5° increased, whereas the femur was 5° internally rotated relative to the pelvis (Table 2). Leg lengthening and FV were negatively correlated with ΔFPP-RCP, with correlation coefficients of 0.376 (*p* < 0.001) and 0354, *p* < 0.001), respectively; these were considered moderately correlated. However, ΔGFO was positively correlated with leg lengthening, with a CC of 0.334 (*p* < 0.001). This was considered a weak correlation (Figure 5a–c, Table 2).

Therefore, leg lengthening showed the highest correlation with ΔFPP-RCP among the parameters studied (Figure 5a–c). Regarding the multiple regression analysis, only leg lengthening and preoperative FPP-PCA were significantly correlated with ΔFPP-RCP, with standardizing coefficients of −0.373 (*p* < 0.001; 95% confidence interval, −0.744 to −0.296) and −0.358 (*p* < 0.001; 95% confidence interval, −0.363 to −0.122), respectively.

### 3.3. Reliability of the Measurement Values

The statistical power of the correlation between ΔFPP-RCP and GFO, ΔFV, and leg lengthening was 0.935. The intra- and interobserver intraclass CCs ranged from 0.816 to 0.902 for all measurements. Adverse events affecting the postoperative course, such as surgical site infection, fracture, and dislocation, were not observed. Moreover, medical complications that could have affected the postoperative course in this study period were not observed.

## 4. Discussion

From our findings, leg lengthening showed the highest correlation with ΔFPP-RCP among the parameters studied. Moreover, leg lengthening and preoperative FPP-RCP were only associated with FPP-RCP in the multivariate analysis. This result is similar to that reported by Uemura et al. [16], who reported that hip rotation following THA was correlated with the amount of leg lengthening, although the FV was not correlated with FPP-RCP in their study. This was presumably due to the soft tissue balance around the hip joint. Hip rotation is defined as the positional relationship between the pelvis and femur. If the anteversion angles were the same as those before surgery, leg lengthening would generate tensile forces in the cephalocaudal direction in the iliotibial tract, gluteus minimus, gluteus medius, psoas, short rotator, and adductor muscles. This may be because the iliotibial tract, gluteus minimus, gluteus medius, iliopsoas, and adductor muscles, except for the short rotators, exerted forces in the direction of internal rotation of the femur relative to the pelvis when the distance between the pelvis and femur was kept constant by these tensile forces. In addition, as the GFO increased, the femur tended to be internally rotated in relation to the pelvis, suggesting that the gluteus minimus, gluteus medius, iliopsoas, and adductor muscles, other than the short rotators, also exerted force in the direction of internal rotation of the femur relative to the pelvis when the GFO increased. In contrast, the femur tended to be internally rotated relative to the pelvis when the FV increased. Kobayashi et al. [17] suggested the existence of a mechanism that adjusts anteversion between the pelvis and femur. We considered that this factor could also be explained by soft tissue balance based on the findings of the current study. Furthermore, leg lengthening was considered the only factor that affected FPP-RCP, which the surgeon could control in planning or during THA because we could not adjust the preoperative FPP-PCA. Therefore, surgeons should consider leg lengthening for suitable hip rotation following THA.

In actual planning, when leg lengthening is increased, increasing stem anteversion may cause internal rotation of the hip joint; consequently, the foot and toes will rotate internally. However, in cases where the hip is internally rotated, it may be better not to excessively increase leg lengthening, decrease anterior stem anteversion, or increase GFO. Physicians should also be cautious when managing patients with highly external rotation in their hip joint as leg lengthening in such cases can induce internal rotation of the hip. Consequently, excessive leg lengthening in these patients could result in large internal rotation, potentially leading to complications such as dislocation due to implant impingement.

Moreover, in the multivariate analysis, leg lengthening was identified as the only factor effecting ΔFPP-RCP that physicians could control. In other words, even if the stem could be inserted as planned, leg lengthening may change the anteversion relative to the pelvis. In the univariate analysis, leg lengthening and FV were equally related to FPP-RCP; however, in the multivariate analysis, only leg lengthening was significantly related to FPP-RCP. This was thought to be due to the confounding effect of the FV with GFO, as stem anteversion affects FO and GFO.

To the best of our knowledge, only a few studies have measured the GFO and leg lengthening using the 3D method similar to this study. When measured with plain radiographs, neither the GFO nor leg lengthening can be accurately assessed because of differences in hip rotation [34,35,40]. Consequently, the values obtained using plain radiographs are not suitable for direct reflection in preoperative planning. In particular, stem anteversion affects the GFO and leg lengthening, and the FPP-RCP can be measured using only 3D measurements. Therefore, our findings are valuable because the factors obtained from plain radiographs were not included.

This study has some limitations. First, the sample size was small (*n* = 124) and we excluded the patients with any symptoms at the thoracic and lumbar spine, knee, or ankle to avoid affecting other postoperative outcomes, which may have led to a bias. Second, this was a single-center, retrospective, cross-sectional study, which may limit the applicability of our findings to other populations. Third, FPP-RCP in this study was measured using preoperative and 1-week postoperative CT images. Although it is not possible to evaluate the alignment with a matched coordinate system unless the images are reconstructed from CT images, it is not practical to perform examinations frequently. Fourth, the CT images were obtained in the supine position; therefore, only alignment evaluation in the supine position could be performed. Hardwick-Morris et al. noted that although the average FPP-PCA rotation from supine to upright was approximately 2°, the variation was large, with many cases varying by ≥10° [41]. Moreover, the CC was not high and was considered moderate. Therefore, we could not survey the association between hip rotation and hip function, thus, we could not provide the acceptable leg lengthening in the current study. Therefore, an exactly controlled large sample size and long-term evaluation are required in the future.

## 5. Conclusions

This study demonstrated that FPP-RCP was only associated with leg lengthening as a factor that physicians could control, as noted in multivariate analysis. In actual planning it is advisable to avoid excessive leg lengthening, decreases to anterior stem anteversion, or increases to the GFO in cases wherein the hip is internally rotated. Moreover, for patients with large external rotation in their hip joint physicians should avoid substantial leg lengthening as it may exacerbate internal rotation of the hip. This misalignment could lead to relative malpositioning, possibly leading to implant impingement and/or dislocation following THA.

## Figures and Tables

**Table 1:** Demographic data of the participants.

Sex (Female/Male)	31/93
Age (years) *	57.26 ± 10.6 (36–78)
Body mass index (kg/m^2^)	24.4 ± 4.2 (17.4–36.8)
Surgical side (right/left)	74/50
Primary disease	DDH: 105 Osteonecrosis of the femoral head: 17 Primary HOA: 2

* Mean ± standard deviation (range). DDH, developmental dysplasia of the hip; HOA, hemilateral hip osteoarthritis.

**Table 2:** Measurement value of pelvic and femoral parameters after total hip arthroplasty.

	Before Surgery	After Surgery	Difference: Δ	*p*-Value
GFO (mm) *	66.7 ± 7.1 (51.8–87.1)	64.2 ± 7.0 (45.6–82.6)	−2.5 ± 6.7 (−22.0–9.9)	<0.001
Leg lengthening (mm) *			10.0 ± 6.3 (−0.2–30.0)	
FV (°)	23.4 ± 13.5 (−15.7–55.2)	26.9 ± 10.9 (−3.8–57.3)	3.5 ± 12.4 (−31.6–53.6)	<0.001
FPP-PCA (°)	3.0 ± 13.0 (−23.2–38.9)	−1.9 ± 11.4 (−31.1–28.1)	−4.9 ± 8.8 (−24.8–13.1)	<0.001

* Mean ± standard deviation (range). The values were adjusted to 100 cm of body height. GFO, global femoral offset; FV, femoral version; FPP-RCP, retrocondylar plane relative to the functional pelvic plane.

## Data Availability

The original contributions presented in this study are included in the article. Further inquiries can be directed to the corresponding author.

## Abbreviations

The following abbreviations are used in this manuscript:

THA	total hip arthroplasty
CT	computed tomography
FPP	functional pelvic plane
RCP	retrocondylar plane
3D	three-dimensional
FV	femoral version
GFO	global femoral offset
FNA	femoral neck anteversion
mHHS	modified Harris hip score
CC	correlation coefficient
OA	osteoarthritis
HOA	hip osteoarthritis

## References

[1] Widmer K.H., Zurfluh B. (2004). Compliant positioning of total hip components for optimal range of motion. J. Orthop. Res..

[2] Malik A., Maheshwari A., Dorr L.D. (2007). Impingement with total hip replacement. J. Bone Jt. Surg. Am..

[3] Lewinnek G.E., Lewis J.L., Tarr R., Compere C.L., Zimmerman J.R. (1978). Dislocations after total hip-replacement arthroplasties. J. Bone Jt. Surg. Am..

[4] Weber M., Woerner M., Craiovan B., Voellner F., Worlicek M., Springorum H.R., Grifka J., Renkawitz T. (2016). Current standard rules of combined anteversion prevent prosthetic impingement but ignore osseous contact in total hip arthroplasty. Int. Orthop..

[5] Eilander W., Harris S.J., Henkus H.E., Cobb J.P., Hogervorst T. (2013). Functional acetabular component position with supine total hip replacement. Bone Jt. J..

[6] Miki H., Kyo T., Sugano N. (2012). Anatomical hip range of motion after implantation during total hip arthroplasty with a large change in pelvic inclination. J. Arthroplast..

[7] Yoshimine F. (2006). The safe-zones for combined cup and neck anteversions that fulfill the essential range of motion and their optimum combination in total hip replacements. J. Biomech..

[8] Nishihara S., Sugano N., Nishii T., Ohzono K., Yoshikawa H. (2003). Measurements of pelvic flexion angle using threedimensional computed tomography. Clin. Orthop. Relat. Res..

[9] Patel A.B., Wagle R.R., Usrey M.M., Thompson M.T., Incavo S.J., Noble P.C. (2010). Guidelines for implant placement to minimize impingement during activities of daily living after total hip arthroplasty. J. Arthroplast..

[10] Pierrepont J., Yang L., Arulampalam J., Stambouzou C., Miles B., Li Q. (2018). The effect of seated pelvic tilt on posterior edge-loading in total hip arthroplasty: A finite element investigation. Proc. Inst. Mech. Eng. H.

[11] DiGioia A.M., Hafez M.A., Jaramaz B., Levison T.J., Moody J.E. (2006). Functional pelvic orientation measured from lateral standing and sitting radiographs. Clin. Orthop. Relat. Res..

[12] Lazennec J.Y., Charlot N., Gorin M., Roger B., Arafati N., Bissery A., Saillant G. (2004). Hip-spine relationship: A radio-anatomical study for optimization in acetabular positioning. Surg. Radiol. Anat..

[13] Pierrepont J., Hawdon G., Miles B.P., O’Connor B., Baré J., Walter L.R., Marel E., Solomon M., McMahon S., Shimmin A.J. (2017). Variation in functional pelvic tilt in patients undergoing total hip arthroplasty. Bone Jt. J..

[14] Lembeck B., Mueller O., Reize P., Wuelker N. (2005). Pelvic tilt makes acetabular cup navigation inaccurate. Acta Orthop..

[15] Imai N., Ito T., Takahashi Y., Horigome Y., Suda K., Miyasaka D., Endo N. (2013). Do Femoral neck and stem anteversion affect final femur rotation and pelvic positioning after total hip arthroplasty?. Open J. Orthop..

[16] Uemura K., Takao M., Hamada H., Sakai T., Sugano N. (2018). Change in axial rotation of the femur in the resting supine position following total hip arthroplasty. Artif. Organs.

[17] Kobayashi D., Choe H., Kobayashi N., Watanabe S., Inaba Y. (2023). Effects of changes in whole-body alignment on ipsilateral knee pain after total hip arthroplasty. J. Orthop. Sci..

[18] McKenna C., Wade R., Faria R., Yang H., Stirk L., Gummerson N., Sculpher M., Woolacott N. (2012). EOS 2D/3D X-ray imaging system: A systematic review and economic evaluation. Health Technol. Assess..

[19] Garg B., Mehta N., Bansal T., Malhotra R. (2020). EOS imaging: Concept and current applications in spinal disorders. J. Clin. Orthop. Trauma.

[20] Buller L.T., McLawhorn A.S., Maratt J.D., Carroll K.M., Mayman D.J. (2021). EOS Imaging is Accurate and Reproducible for Preoperative Total Hip Arthroplasty Templating. J. Arthroplast..

[21] Cho E., Hisatome T., Oda S., Fujimaki H., Nakanishi K. (2022). Accuracy of acetabular cup placement during anterolateral supine total hip arthroplasty using intraoperative fluoroscopy: A retrospective study. J. Orthop. Surg. Res..

[22] Stolarczyk A., Stolarczyk M., Stępiński P., Dorocińska M.K., Świercz M., Szymczak J., Żarnovsky K., Żuchniewicz A., Maciąg B.M. (2021). The direct anterior approach to primary total hip replacement: Radiological analysis in comparison to other ap-proaches. J. Clin. Med..

[23] Ohta Y., Sugama R., Minoda Y., Mizokawa S., Takahashi S., Ikebuchi M., Nakatsuchi T., Nakamura H. (2022). Is the anterolateral or posterolateral approach more effective for early postoperative recovery after minimally invasive total hip arthroplasty?. J. Clin. Med..

[24] Sugano N., Nishii T., Miki H., Yoshikawa H., Sato Y., Tamura S. (2007). Mid-term results of cementless total hip replacement using a ceramic-on-ceramic bearing with and without computer navigation. J. Bone Jt. Surg. Br..

[25] Murray D.W. (1993). The definition and measurement of acetabular orientation. J. Bone Jt. Surg. Br..

[26] Suda K., Ito T., Miyasaka D., Imai N., Minato I., Endo N. (2016). Cup implantation accuracy using the HipCOMPASS mechanical intraoperative support device. SpringerPlus.

[27] Imai N., Takubo R., Suzuki H., Shimada H., Miyasaka D., Tsuchiya K., Endo N. (2019). Accuracy of acetabular cup placement using CT-based navigation in total hip arthroplasty: Comparison between obese and non-obese patients. J. Orthop. Sci..

[28] Matsubayashi S., Isobe Y., Chiba K., Tsujimoto R., Osaki M., Imamura T., Tsurumoto T. (2021). Measurement of femoral axial offset. J. Orthop. Res..

[29] Dorr L.D., Malik A., Dastane M., Wan Z. (2009). Combined anteversion technique for total hip arthroplasty. Clin. Orthop. Relat. Res..

[30] Nozaki A., Imai N., Funayama K., Horigome Y., Suzuki H., Minato I., Kobayashi K., Kawashima H. (2023). Accuracy of ZedView, the software for three-dimensional measurement and preoperative planning: A basic study. Medicina.

[31] Ariumi A., Sato T., Kobayashi K., Koga Y., Omori G., Minato I., Endo N. (2010). Three-dimensional lower extremity alignment in the weight-bearing standing position in healthy elderly subjects. J. Orthop. Sci..

[32] Sato T., Koga Y., Omori G. (2004). Three-dimensional lower extremity alignment assessment system: Application to evaluation of com-ponent position after total knee arthroplasty. J. Arthroplast..

[33] Kubota S., Inaba Y., Kobayashi N., Choe H., Tezuka T., Saito T. (2017). Comparison of improved range of motion between cam-type femoroacetabular impingement and borderline developmental dysplasia of the hip -evaluation by virtual osteochondroplasty using computer simulation. BMC Musculoskelet. Disord..

[34] Hirano Y., Imai N., Nozaki A., Horigome Y., Suzuki H., Kawashima T. (2023). The association of postoperative global femoral offset with total hip arthroplasty outcomes. Sci. Rep..

[35] Imai N., Hirano Y., Endo Y., Horigome Y., Suzuki H., Kawashima H. (2024). The sum of the leg length discrepancy and the difference in global femoral offset is equal to that of the contralateral intact side and improves postoperative outcomes after total hip arthroplasty: A three-dimensional analysis. J. Clin. Med..

[36] Sugano N., Noble P.C., Kamaric E. (1998). A comparison of alternative methods of measuring femoral anteversion. J. Comput. Assist. Tomogr..

[37] Guilford J.P., Fruchter B. (1973). Correlation. Fundamental Statistics in Psychology and Education.

[38] Dancey C.P., Reidy J. (2007). Statistics Without Maths for Psychology.

[39] Haldun A. (2018). User’s guide to correlation coefficients. Turk. J. Emerg. Med..

[40] Tone S., Hasegawa M., Naito Y., Wakabayashi H., Sudo A. (2022). Comparison between two- and three-dimensional methods for offset measurements after total hip arthroplasty. Sci. Rep..

[41] Hardwick-Morris M., Twiggs J., Kacker K., Miles B., Balakumar J. (2022). Functional femoral anteversion: Axial rotation of the femur and its implications for stem version targets in total hip arthroplasty. Arthroplast. Today.

